# Supplementary material for: HyperGen: compact and efficient genome sketching using hyperdimensional vectors
Source: Bioinformatics. 2024 Jul 16;40(7):btae452. doi: 10.1093/bioinformatics/btae452 (PMC11281827; doi:10.1093/bioinformatics/btae452)
Supplement: btae452_Supplementary_Data [file btae452_supplementary_data.pdf]

Bioinformatics
doi.10.1093/bioinformatics/xxxxxx
Advance Access Publication Date: Day Month Year
Manuscript Category

OXFORD

Supplementary Materials

HyperGen: Compact and Efficient Genome Sketching using Hyperdimensional Vectors

Weihong Xu[1,*], Po-Kai Hsu[2], Niema Moshiri[1], Shimeng Yu[2], and Tajana Rosing[1]

[1]Department of Computer Science and Engineering, University of California San Diego, La Jolla, CA 92093, USA.
[2]School of Electrical and Computer Engineering, Georgia Institute of Technology, Atlanta, GA 30332, USA.

[*]To whom correspondence should be addressed.

Abstract

This document summarizes the supplementary materials.

List of Supplementary Materials

- **Supplementary Table** 1
  Names, versions, and commands of benchmarked genome tools for ANI calculation. The sketch-based tools include: Mash, Dashing 2, and HyperGen. The mapping-based tool is FastANI. The alignment-based tool is ANIm.
- **Supplementary Table** 2
  Specifications (name, description, data size, query genome, and sources) of evaluated datasets.
- **Supplementary Table** 3
  Error metrics for the 100×100 pairwise Jaccard estimation. HyperGen-2048 and HyperGen-4096 use $D = 2048$ and $D = 4096$, respectively. Other tools use their default parameters.
- **Supplementary Figure** 1
  The illustration of HV orthogonality in HyperGen for HV dimension $D = 64$ to 8192 and number of elements $n = 32$ to 256.
- **Supplementary Figure** 2
  The value distribution of sketch hypervectors (HVs) generated by HyperGen when using various scaled factor $S = 800$ to 2000.
- **Supplementary Figure** 3
  The execution time breakdown of HyperGen during genome sketching. The HV dimension ranges from $D = 1024$ to 8192. The HV aggregation optimized by Single Instruction Multiple Data (SIMD) incurs negligible overhead as compared to the FracMinHash step.
- **Supplementary Figure** 4
  The ANI estimation error distribution of database search for all benchmarking tools (HyperGen, Mash, Bindash, Dashing 2, FastANI, and Skani).
- **Supplementary Figure** 5
  The relationship between completeness and absolute ANI estimation error for database search using HyperGen with parameters $k = 21, D = 4096, S = 1500$. The completeness is calculated by BUSCO (https://busco.ezlab.org/). HyperGen achieves smaller ANI estimation error for more complete genomes.

© The Author 2024. Published by Oxford University Press. All rights reserved. For permissions, please e-mail: journals.permissions@oup.com

Table 1. Names, versions, and commands of benchmarked genome tools for ANI calculation. The sketch-based tools include: Mash, Dashing 2, and HyperGen. The mapping-based tool is FastANI. The alignment-based tool is ANIm.

Tool	Version	Commands and arguments
HyperGen	v0.2.2	hyper-gen sketch -D cpu -t 16 -k 21 -s 1500 -d 4096 -p (fna_path) -o (file_out)<br>hyper-gen sketch -D gpu -t 16 -k 21 -s 1500 -d 4096 -p (fna_path) -o (file_out)<br>hyper-gen dist -t 16 -r (ref_sketch) -q (query_sketch) -o (dist_file)
Mash	v2.3	mash sketch (data set) -o (sketches) -p 16<br>mash dist (query genome) (sketches) -p 16
Bindash	v1.0	bindash sketch –nthreads=16 –listfname=(genome_list) –outfname=(sketch)<br>bindash dist (query_sketch) (ref_sketch) –nthreads=16 –outfname=(dist_out)
Sourmash	v4.5	sourmash sketch dna –output-dir (sketches) (data set)<br>sourmash compare (sketches)/*.sig -k 21 –max-containment –ani
Dashing 2	v2.1.19	dashing2 sketch –bagminhash -k 21 -S (sketch_size) -p 16 -F (file_list)<br>dashing2 sketch –bagminhash –cache -k 21 -S (sketch_size) -p 16 -F (ref_file) -Q (query_file)<br>dashing2 sketch –set –cache -k 21 -p 16 -F (file_list) –cmpout (file_out)
Skani	v0.2.1	skani sketch -t 16 -c 70 -m 1000 -l (genome_list) -o (sketches)<br>skani dist -t 16 -q (query_sketches) -r (ref_sketches) -o (file_out)
FastANI	v1.33	fastANI –rl (genome_list) -q (query_genome) -t 16
ANIm (nucmer)	v0.2.12	average_nucleotide_identity.py -m ANIm –workers 16 -i (genomes) -o (output_folder)

Table 2. Detailed specifications for the evaluated genome datasets.

Dataset Name	Description	Size	Query Genome	Source
Bacillus cereus	Draft genome assemblies of *Bacillus cereus s.l.* from the prokaryote section of the NCBI Genome database.	3.1GB	*Bacillus anthracis* (NZ_CM002395)	Dataset 2 at http://enve-omics.ce.gatech.edu/data/fastani
Escherichia coli	Draft genome assemblies of *Escherichia coli* from the prokaryote section of the NCBI Genome database.	22GB	*Escherichia coli* (GCA_000303255)	Dataset 3 at http://enve-omics.ce.gatech.edu/data/fastani
NCBI RefSeq	Prokaryotic genomes downloaded from RefSeq database.	5.6GB	*Escherichia coli* K12 W3110 (NC_007779)	Dataset 1 at http://enve-omics.ce.gatech.edu/data/fastani
Parks MAGs	A large collection of metagenome-assembled genomes.	20GB	Pseudomonas stutzeri (Parks GCA_002292085_1)	Dataset 5 at http://enve-omics.ce.gatech.edu/data/fastani
GTDB MAGs	A phylogenetically consistent and rank normalized genome-based taxonomy for prokaryotic genomes sourced from the NCBI Assembly database.	203GB	*Escherichia coli* K12 W3110 (NC_007779)	Release r207 at https://gtdb.ecogenomic.org/

Table 3. Error metrics for the 100×100 pairwise Jaccard estimation. HyperGen-2048 and HyperGen-4096 use $D = 2048$ and $D = 4096$, respectively. Other tools use their default parameters. The ground truth values of Jaccard index are calculated using Dashing 2's exact mode. The command is given in Supplementary Table 1 (The 3rd line of Dashing 2's commands).

Dataset: *Bacillus cereus*			
Tool	MAE ↓	RMSE ↓	MPAE ↓
Mash	0.008	0.011	3.860
Bindash	0.007	0.010	3.748
Dashing 2	0.011	0.016	6.458
Sourmash	0.004	0.005	3.123
HyperGen-2048	0.010	0.013	5.815
HyperGen-4096	0.007	0.009	4.274

Dataset: *Escherichia coli*			
Tool	MAE ↓	RMSE ↓	MPAE ↓
Mash	0.009	0.011	3.500
Bindash	0.007	0.009	2.498
Dashing 2	0.032	0.051	8.709
Sourmash	0.013	0.014	4.394
HyperGen-2048	0.010	0.013	3.267
HyperGen-4096	0.009	0.011	2.641

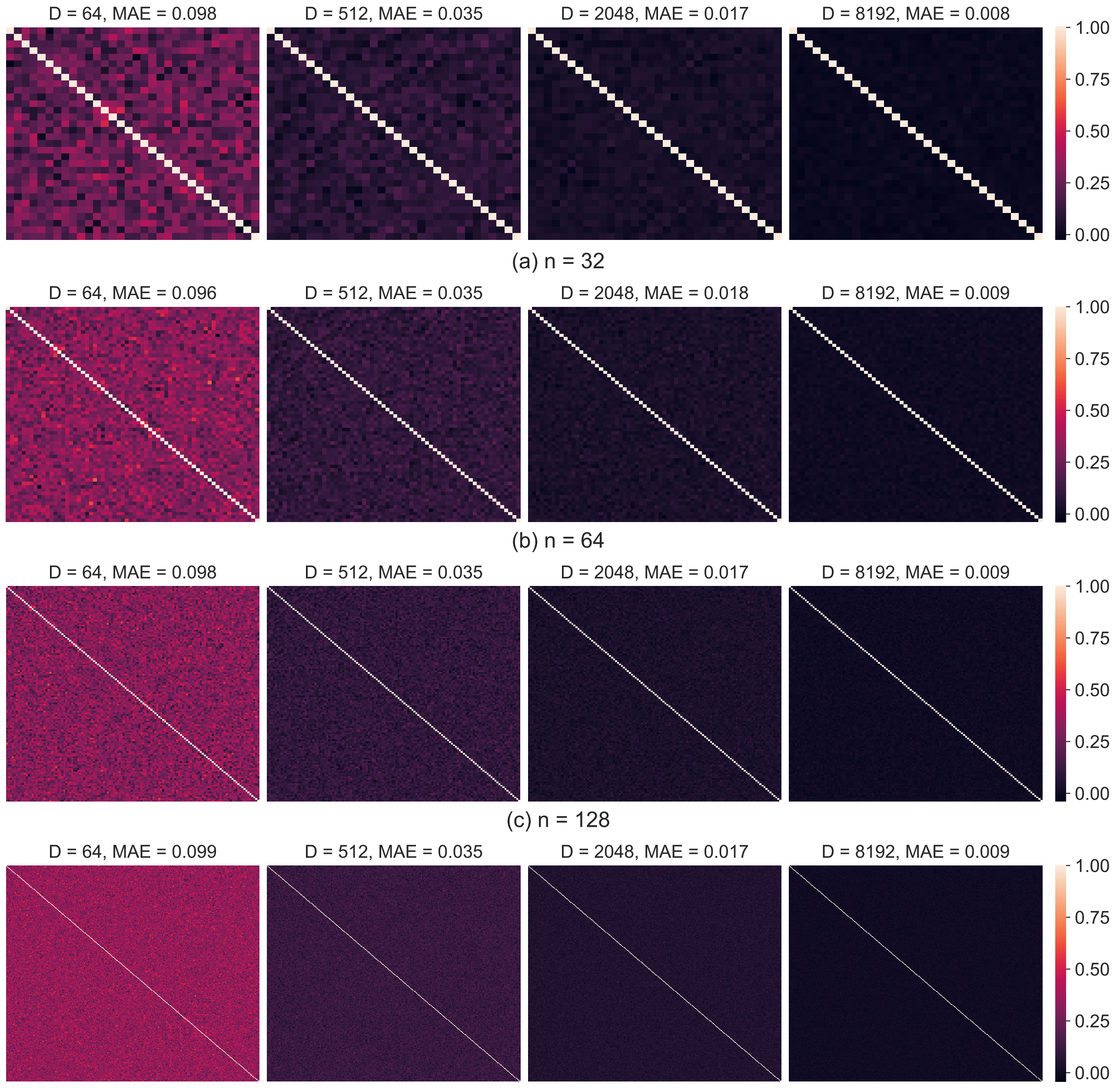

(a) n = 32

(b) n = 64

(c) n = 128

(d) n = 256

Fig. 1: The illustration of HV orthogonality in HyperGen for HV dimension $D = 64$ to 8192 and number of elements $n = 32$ to 256. The pairwise similarity for each HV is computed and depicted. HVs with the same index has similarity close to 1 while HVs with different indices are quasi-orthogonal (similarity close to 0). The mean absolute error (MAE) between the pairwise matrix and identity matrix is computed to measure the quasi-orthogonality. Larger HV dimensions provide better orthogonality (smaller MAEs).

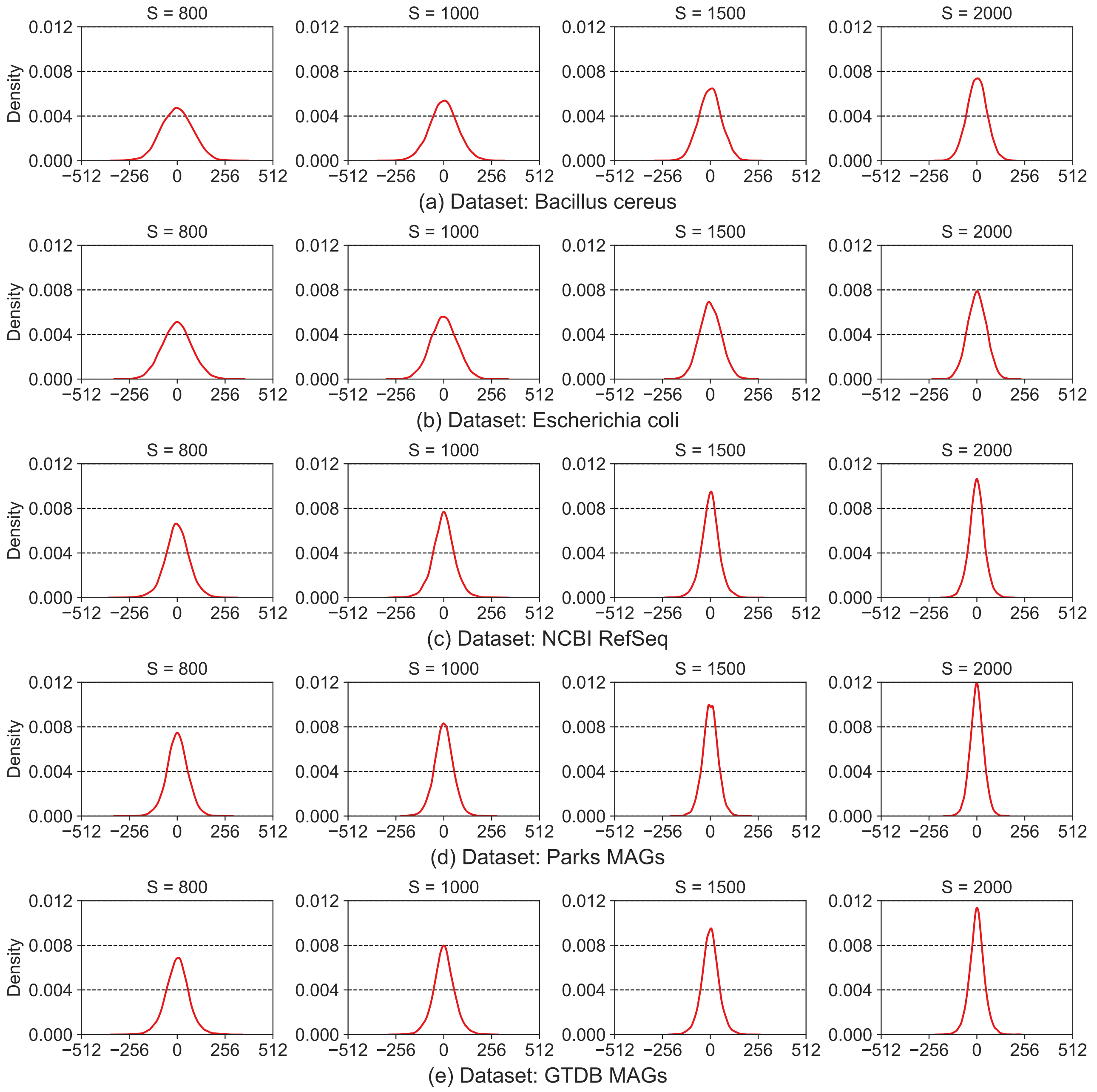

Fig. 2: The value distribution of sketch hypervectors (HVs) generated by HyperGen when using various scaled factor $S = 800$ to 2000. HV values exhibit a bell curve distribution, where the majority of values locate within the range -300 to 300. Sketch HVs can be effectively quantized to about 10 bits without precision loss.

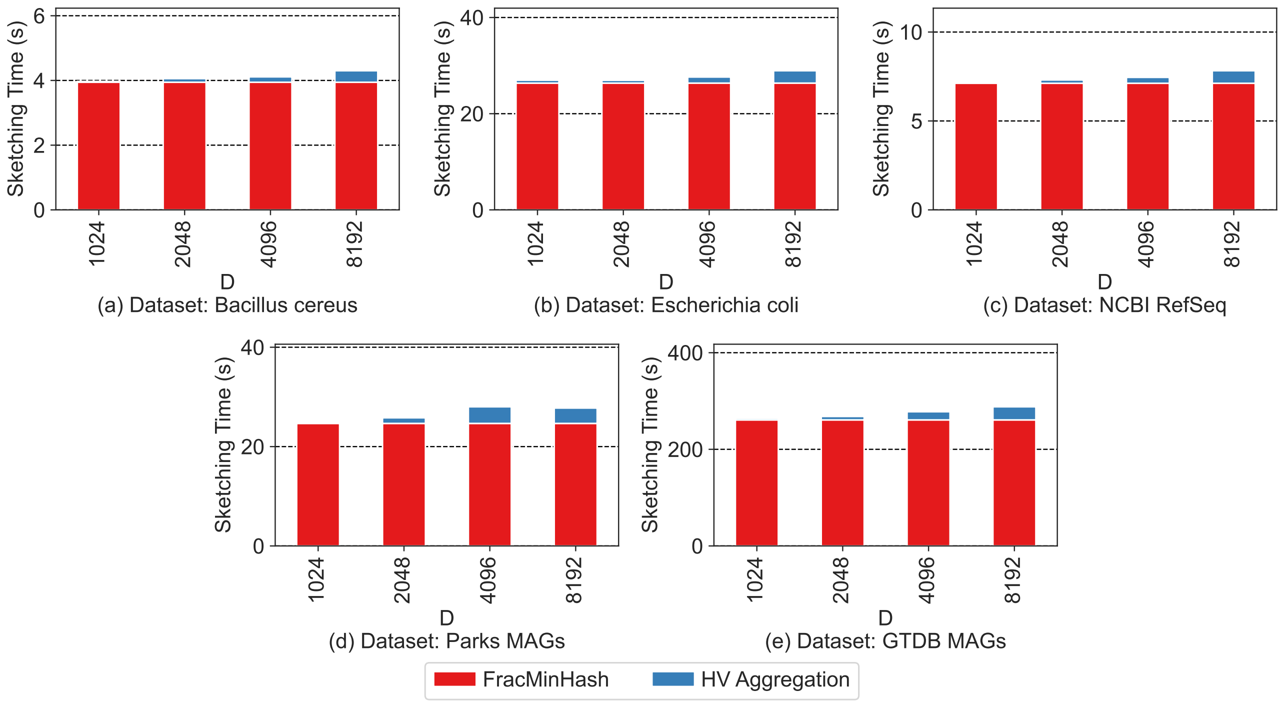

Fig. 3: The execution time breakdown of HyperGen during genome sketching. The HV dimension ranges from $D = 1024$ to 8192. The HV aggregation optimized by Single Instruction Multiple Data (SIMD) incurs negligible overhead as compared to the FracMinHash step.

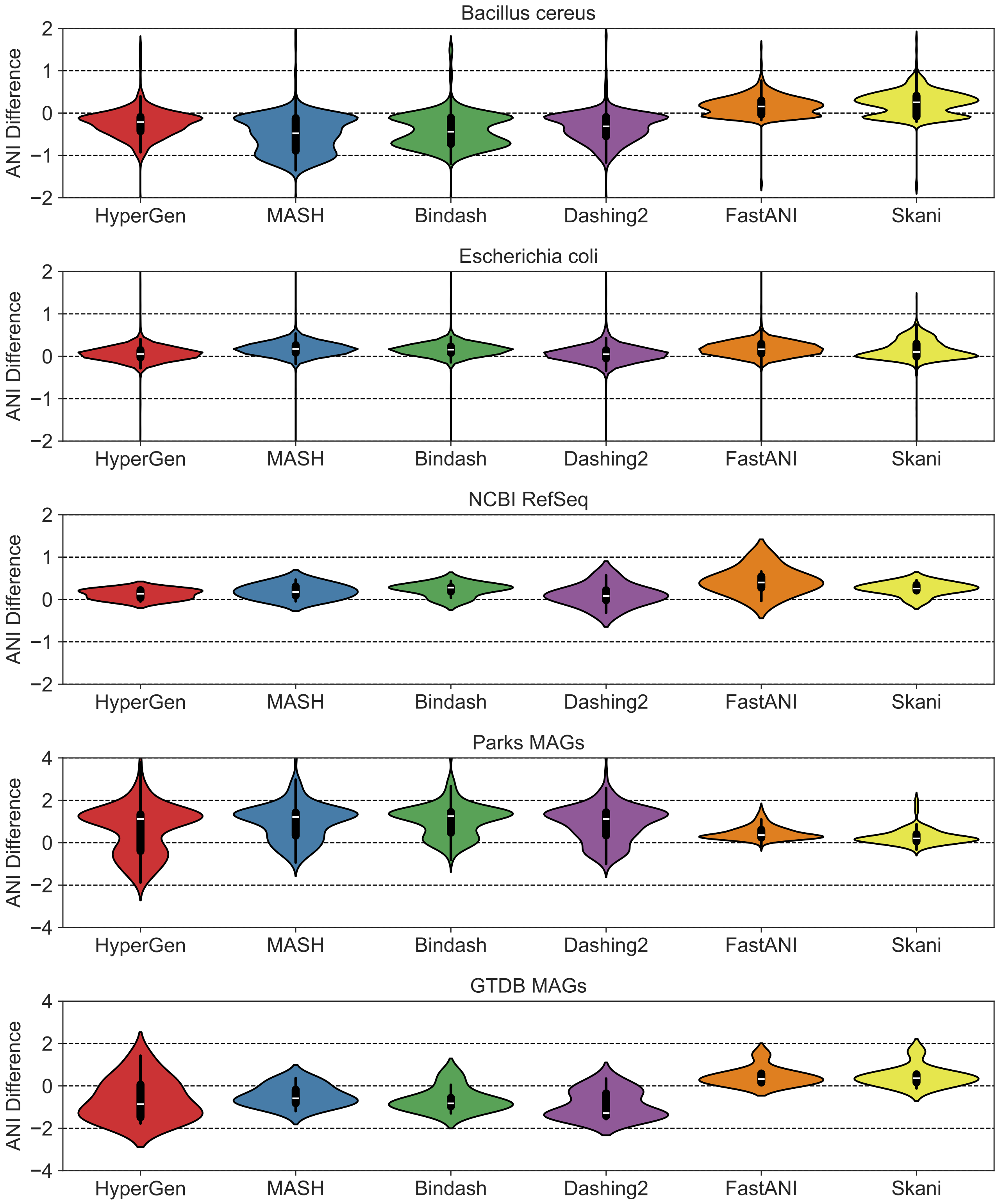

Fig. 4: The ANI estimation error distribution of database search for all benchmarking tools (HyperGen, Mash, Bindash, Dashing 2, FastANI, and Skani). Data points with ANI > 85 are considered here.

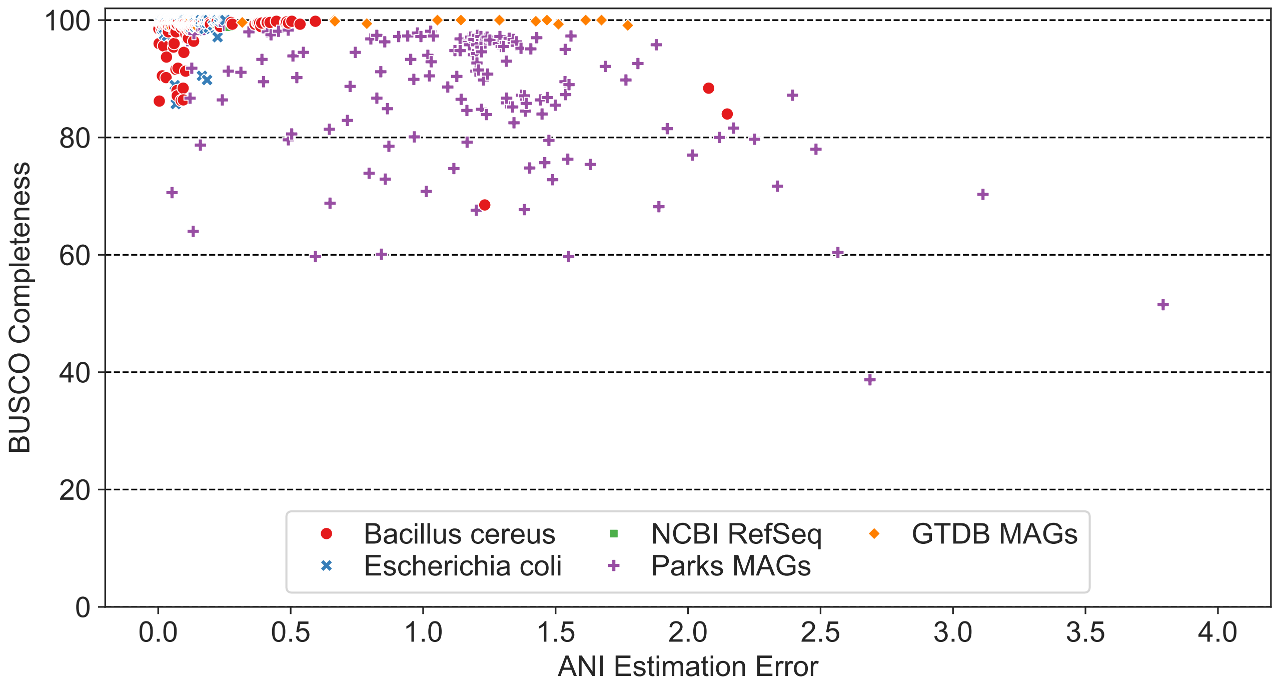

Fig. 5: The relationship between completeness and absolute ANI estimation error for database search using HyperGen with parameters $k = 21$, $D = 4096$, $S = 1500$. The completeness is calculated by BUSCO (https://busco.ezlab.org/). HyperGen achieves smaller ANI estimation error for more complete genomes. Data points with ANI > 85 are considered here.
